# Department of Surgery

**Published:** 1901-06

**Authors:** W. E. A. Wyman, J. A. Sloan, Robert Jay

**Affiliations:** Milwaukee, Wis.; San Francisco, Cal.; West Liberty, Ia.


### DEPARTMENT OF SURGERY.

By W. E. A. Wyman, M.D.V., V.S.,
MILWAUKEE, WIS.

J. A. Sloan, B.Sc., M.D.V.,
SAN FRANCISCO, CAL.

Robert Jay, M.D.V.,
WEST LIBERTY, IA.

### Hoof Diseases.

(Continued from page 303.)

### Hemorrhagic Pododermatitis.

By it is understood an aseptic inflammation of the pododerm, either acute or chronic, circumscribed or diffused, varying in intensity, attacking preferably the posterior section of the hoof. The causes of hemorrhagic pododermatitis are closely related to those of serous pododermatitis, but are more violent, since either a direct hemorrhage or a hemorrhagic exudate follows them. Both the shod and bare hoof are exposed to it, and, here again, the hoof of the anterior extremities and of them the internal face, and especially the pododerm lining the heels and parts related thereto, are predisposed. As in all hoof diseases discussed previously, predisposing and external causes must be considered. Thus faulty hoofs and malpositions of the limb and a disturbed foot axis belong to the predisposing factors, primarily the forward deviations, as camped in front; those which toe-out; the thin-shelled and acute angled hoof; those hoofs affected with contracted quarters and coronary contraction, and, finally, the wry hoof.

Of the external causes mechanical insults play the most important rôle, but, as previously said, they must be of a more violent nature than those producing serous pododermatitis. As far as the balance is concerned, the reader is referred to the chapter on serous pododermatitis.

Predisposing causes, plus external insults of a continuous type, lead eventually to chronic hemorrhagic pododermatitis. According to Eberlein, hemorrhagic pododermatitis, as a rule, starts in the vascular layer, spreading from this to all layers, with a tendency to reach the superficial ones. At first there is a hyperæmia followed by a hemorrhagic inflammatory exudate, with cellular infiltration and depending on the severity of the lesion of the walls of the veins, arteries, and especially the capillaries. A large number of red blood-corpuscles emigrate or blood themselves—that is, a regular hemorrhage occurs. This hemorrhagic exudate and cellular infiltration are followed by an inflammatory œdema of the pododerm, which, by its pressure upon the bloodvessels, brings about anæmia of the parts. In those cases where the exudate reaches the *rete Malpighii* the union between the fleshy and horny laminæ is severed by reason of the fact that the cells of the *rete Malpighii* imbibe the exudate and swell. The œdematous state of the pododerm is best recognized by the slightly fluctuating swelling over the heels.

One of the possible terminations of acute hemorrhagic pododermatitis is resolution, which starts in the vascular layer. As the results of the imbibition of the hemorrhagic exudate by the horn cells and the filling of the horn tubes, with the same, by capillary attraction, one perceives a red, glistening, transparent, soft horn. Chronic inflammation is another termination. Here we see distinct changes of the pododerm which becomes sclerotic; keraphyllocele may also form for the same reasons as in chronic serous pododermatitis. Pressure atrophy, and osteitis of the os pedis, a tree-bark-like appearance, and ringed state of the horny capsule are also observed. The diagnosis of hemorrhagic pododermatis is easy. There is pronounced supporting leg-lameness, which increases on hard ground and going down hill for reasons already repeatedly given. In those cases where the pododerm in the region over the heels is involved the animal knuckles over while moving. In the standing posture the animal points forward and moves the hoof to and fro with volar flexion of the phalangeal articulations when the pododerm of the bulbs is diseased. Palpation with the percussion hammer or pincers produces decided pain; palpation of the diseased hoof with the hand shows increased heat of the hoof. The œdematous state of the inflamed pododerm, of course, is only visible in the region of the coronet and over the heels, while subcutaneous

œdema, at times extending half-way up the shin, depending on the disturbed circulation of the hoof, is seen. Palpation of the digital artery exhibits a full, bounding pulse.

The decisive symptom at the same time is met with when the hoof is pared out. As soon as the dry horn has been removed the diagnostic glistening, moist, diffused, red state of the horn, most frequently encountered at the angle of the bars, becomes noticeable. In chronic hemorrhagic pododermatitis the same redness is observed as well as the other symptoms already discussed under chronic serous pododermatitis. In deeply pigmented hoofs the reddish state of the horn in either acute or chronic hemorrhagic pododermatitis may be impossible to recognize. From a differential stand-point the following points are of special interest:

Corns. The discoloration of the horn is circumscribed, while in hemorrhagic pododermatitis it is diffused, spreading to the neighboring parts.

Serous pododermatitis. There is a yellow discoloration and absence of a full, bounding hoof pulse.

Purulent pododermatitis. Palpation exhibits a circumscribed intensely painful area, while inspection reveals a black circumscribed or punctiform field.

(To be continued.)

